# Bullying in schools: prevalence, bystanders’ reaction and associations with sex and relationships

**DOI:** 10.1186/s40359-021-00685-5

**Published:** 2021-11-22

**Authors:** Temesgen Demissie Eijigu, Seleshi Zeleke Teketel

**Affiliations:** 1 Department of Psychology, Institute of Educational and Behavioral Sciences, Debre Markos University, Debre Markos, Ethiopia; 2 School of Psychology, Addis Ababa University, Addis Ababa, Ethiopia

**Keywords:** Defending, Passive bystanding, Bullying, Bystander reaction

## Abstract

**Background:**

Bullying and peer victimization are the most pressing social problems affecting the wellbeing of children and adolescents. This study attempts to estimate the prevalence and examine the association of bystander’s sex, her/his relationship with the victim and with the bully, and bystander’s reaction to school bullying in East Gojjam Administrative Zone, Ethiopia.

**Methods:**

This study followed an explanatory mixed-method study design. For the quantitative phase, 612 participants were selected using multistage cluster sampling techniques and for qualitative phase, 18 participants were selected using purposive sampling technique. We used self-reported questionnaires and semi-structured interviews to collect data from students attending grades 7, 8, 9, and 10.

**Results:**

This study revealed that 55% of bystanders remained passive while 38% of them defended the victim upon witnessing bullying incidents. Pearson Chi-Square test for independence indicated a significant association between bystanders’ relationship with the victim and/or bully, and bystanders’ reaction. In contrast, sex has no significant association with bystanders’ reaction. The semi-structured interview data also suggested that large number of bystanders most often stood by passively while some of them defended the victim.

**Conclusion:**

The practice of defending among students attending their education in governmental primary and secondary schools in East Gojjam Administrative Zone was low. Close social relationships (being close friends, relatives, and classmates) with the victim and bully were significantly associated with the practice of defending.

## Background

Bullying and peer victimization are the most pressing social problems affecting the wellbeing of children and adolescents [1]. Although bullying occurs in many contexts [2], it is predominantly prevalent within a school setting [3, 4]. For instance, over 90% of primary and secondary school students in Australia witnessed verbal bullying, and more than 60% witnessed physical bullying in their schools [5]. Moreover, a study on the prevalence of being bullied in South Australian schools depicted that approximately one of every two secondary school students experienced victimization by peers while at school [3].

The problem of violence and bullying is also prevalent in Ethiopia [6–10]. A study in Addis Ababa revealed that 84% of teachers and directors confirmed that violence is a problem in and around primary and secondary schools, mainly targeting girls and smaller children [7]. Similarly, a national study in Ethiopia revealed that 13.1% and 16.7% of children have been left out and hit by other children, respectively, in their class [10].

The situation of school bullying in the East Gojjam Zone does not seem an exception. For example, in the 2014 academic year, more than 57% of students in Menkorer High School at Debre Markos Town, the capital of East Gojjam Administrative Zone, experienced physical and sexual violence [11].

School bullying is viewed as a group phenomenon that, in addition to bullies and victims, involves a large number of bystanders who witness bullying [12–14]. For instance, two studies in Canada illustrated that peer bystanders were present in more than five out of six bullying episodes [13, 14]. Another natural observational research also reported that peers were present closely in nine out of ten bullying episodes [13]. Although bullying often occurs in the presence of large bystanders who have a high potential to reduce it, most do not intervene to stop it [13, 14].

In bullying situations, bystanders may take the following four roles: (1) assistants, who join in the bully’s side (2) reinforcers, who encourage bullies (3) passive bystanders, who merely watch what is happening and (4) defenders, who stand up on behalf of victims [12]. Recent studies proposed three forms of bystander roles as passive bystanders, defenders, and pro-bully/bully supporters/by combining the roles of assistant and reinforcers [15].

A study in 1220 elementary school children from grades four to six found that low scores on the anti-bullying attitude scale were associated with bullying, assisting the bully, and reinforcing the bully. In contrast, high scores on that scale were related to defending the victim and remaining passive in bullying situations [16]. Since passive bystanders scored high in anti-bullying attitude and moral disapproval scores of bullying, it is easier to change them to the defenders than assistants and reinforcers. Thus, passive bystanders were the focus of this study. Besides, passive bystanders and defenders account for more than half of the bystanders who could play a key role in reducing bullying. To our knowledge, no previous studies in Ethiopia estimated the extent of defenders and passive bystanders during bullying in primary and general secondary school students. Thus, one of the focuses of this study was to estimate the extent of defending and passive bystanding behaviors during school bullying.

Empirical findings reported gender differences in defending and passive bystanding behavior [5, 17–20]. Several studies revealed that girls are more involved in defending the victim [16, 17, 20–22] and remaining passive in bullying situations than boys, whereas boys were more involved in supporting bullies as assistants and reinforcers than girls [16, 17, 20, 23]. In addition, some studies have shown a significant association between the gender of the bystander, the gender of the bully, and the victim [13]. Their findings suggest that boys are more likely to defend when the bully or victim is male, whereas girls are more likely to defend when the bully or victim is female. Likewise, some studies [24, 25] documented that students were more likely to defend their same-sex peers than opposite-sex peers. This shows that previous studies emphasized sex differences and how bystanders are more likely to help the same sex victim [17–19]. They did not answer the question, “To what extent do female and/or male bystanders defend or remain passive upon witnessing a girl victimizing a boy, a boy victimizing a boy, a boy victimizing a girl, and a girl victimizing a girl. Thus, further research is needed to fill these knowledge gaps.

Furthermore, bystanders’ relationships with the victim or bully may also influence defending or passive bystanding behavior [26, 27]. These studies revealed that bystanders who had a close relationship with the victim are more likely to help the victim, whereas those who had a close relationship with the perpetrator and no relationship with the victim are more likely to remain passive; sometimes it may even initiate co-bullying [26]. The motives for co-bullying or non-intervention, were reported to come from fear of friendship loss, perceived peer pressure, or to not disprove the actions of friends.

In the culture of Amhara, when one's close relative or friend is attacked, he/she will not watch the incident passively. At least, he/she is expected to separate the bully and the victim. This strong social bond among Amhara society [28] makes it reasonable to include bystanders’ relationship with the bully and victim in the study.

## Research question

This research planned to answer the following questions:

- To what extent do students defend or remain passive during bullying incidents in primary and secondary schools in East Gojjam Administrative Zone?
- To what extent do male and female bystanders defend or remain passive upon witnessing a boy victimizing a boy, a boy victimizing a girl, a girl victimizing a girl, and a girl victimizing a boy?
- Does the relationship between the bystander and the victim or the bystander and the bully make a difference in the bystander’s reaction?

## Methods

### Aim

This study aimed to estimate the prevalence and examine the association between bystander’s sex, her/his relationship with the victim and with the bully, and bystander’s reaction to school bullying in East Gojjam Administrative Zone, Ethiopia.

### Study design

This study followed an explanatory sequential mixed methods design [29, 30] with quantitative data collection and analysis in the first phase and qualitative data collection and analysis in the second phase. Mixed methods design was selected to other designs since the complex nature of bystanding behaviors during school bullying requires an investigation from multiple ways.

### Setting

The study was conducted in primary and general secondary schools from Aneded, Debre Markos, Enebesie Sar Medir, Enemay, and Machakel Woredas of East Gojjam Administrative Zone, Amhara National Regional State, Ethiopia. These five Woredas consists of 181 second cycle primary schools (Grades 5–8) and 19 secondary schools (grades 9 and 10). Primary and general secondary schools from Woredas in East Gojjam Administrative Zone were selected due to bullying prevalence and its serious consequences. In addition to familiarity with the language and culture, the researcher works in the study area that may contribute to the study.

### Participants and sampling techniques

The quantitative data were drawn from 612 students aged 12–16 years attending five primary schools in grades 7and 8 and five general secondary schools in grades 9 and 10 (see Table 1). To select participants for this study, we used a multistage cluster sampling procedure. In the first stage, we subdivided the 19 Woredas of East Gojjam Administrative Zone into five groups based on the number of students’ population from grades 7–10. From each group, we selected one woreda randomly. Then, from each woreda, one general secondary school was chosen randomly. Next, for accessibility and comparison purposes, from all primary schools in the area where the selected general secondary schools were situated, one primary school from each woreda was selected by using lottery method. Then, one class from each grade in each school was selected by applying lottery method. Accordingly, 20 classes of students from both primary and general secondary schools (10 classes each) were invited to participate in the study.

**Table 1:** Participants’ demographic characteristics

Variables	Frequency	Percent
*Sex*		
Male	266	43.5
Female	346	56.5
≤ 13	70	11.4
14	137	22.4
15	142	23.2
16	263	43.0
*Grade level*		
Grade 7	175	28.6
Grade 8	174	28.4
Grade 9	155	25.3
Grade 10	108	17.6
*Residence*		
Rural	249	40.7
Urban	363	59.3
*Number of close friends*		
0	4	0.7
1–2	190	31.0
3–4	265	43.3
5–6	105	17.2
7 and above	48	7.8

On the other hand, the qualitative data were drawn from 18 participants (9 boys and 9 girls) who witnessed bullying incidents. To select participants, a purposive sampling technique was employed. With the help of school principals, homeroom teachers, and classroom representatives, students who usually defend or passively watch when witnessing bullying incidents were selected. Participants’ age ranged from 14 to 16 years, and more than 22% were from rural areas. Concerning grade level, five students were from grade seven, four students from grade eight, five students from grade nine, and four students from grade ten.

### Inclusion and exclusion criteria

All students who were attending grades 7–10 education in 20 classes were included in the study. Those students who witnessed bullying were also included in the study. Those students outside the age range of 12–16 years, who did not witness bullying, and absent from class during data collection were excluded from the study.

### Data collection instruments

#### Questionnaire

To collect quantitative data, self-report questionnaires have been adapted from previous sources [17]. To estimate the prevalence and examine the association between bystander’s sex, her/his relationship with the victim and with the bully, and bystander’s reaction to school bullying, participants were asked to recall one particular incident where they witnessed a student/s bullying another student since the beginning of this semester. The items included in the questionnaire were: “Describe in brief the nature of the bullying incident you witnessed,” “When and where the bullying incident happened,” “Describe the characteristics of the victim and the bully (sex, grade, bystander’s relationship with the victim/bully such as close relative, close friend, classmate, a person that I knew but have no close relationship, or person that I did not know),” and “What did you do when you witnessed bullying incident?”.

A bystander was placed into categories of defender, passive bystander, and bully supporter based on his/her reactions to the bullying incident in the school:

1. If a student answers, “I joined in the bullying when the bully had started it,” “I assisted the bullying by doing something for the bully”, and/or “I giggled, laughed, shouted, or made similar reactions,” s/he is categorized under “bully supporter.”
2. If a student answers, “I kept looking at the bullying without siding anyone,” “Nothing, I went away from the situation,” and/or “Nothing, I pretended not to notice what was happening,” s/he is categorized under “passive bystander.”
3. If a student answers, “I tried to help in some way but was not successful,” and/or I tried to help in some way and was successful,” s/he is categorized under “defender.”

The English version of the instrument was translated into the Amharic language by three language experts who have Ph.D. in Teaching Amharic, Linguistic, and Teaching English as a Foreign Language and whose mother tongue was Amharic. The principal investigator of this study synthesized a single version by combining the best cultural translation of each item. The appropriateness of the synthesized translated version was judged by three language experts (two Amharic, one English) and two psychologists. By taking into account the feedback offered by professionals, in view of the study's objectives and reviewed literature, the researcher of this study revised the synthesized translated version of the instrument. An expert from Debre Markos University who had a doctoral degree in Teaching English as a Foreign Language back-translated the synthesized version from Amharic into English. Moreover, the Amharic version of the instrument was submitted to seven psychology instructors of Debre Markos University to assess the instruments' content validity. Based on comments of experts, some items were modified. Finally, the questionnaire was administered to the participants during the period 01–31 January 2019.

#### Semi-structure interview

The interviews were conducted face to face by the principal investigator from 01 April to 02 May 2019 using semi-structured open-ended items with probing questions. Interviews were conducted at the offices of the counselor, or school director lasted between 30 and 45 min. Students were alone (not accompanied by guardians/parents) when interviews were administered. All interviews were audio-recorded, transcribed, and notes were taken properly. Items in the interview guide include: “If you have witnessed someone being bullied by another student, tell me what happened?”, “How did you feel when you saw bullying happening?”, “What did you do when you witnessed bullying happening? Why?”, “Who else witnessed the bullying situations besides you?”, “What did they do when this was happening?”, “Why do you think they reacted this way?”, “Why do you think that some students defend and others remain silent in bullying incidents?” and “How do you describe boys and girls' engagement in defending or passive bystanding behaviors?”.

#### Data analysis techniques

Researchers employed percentage to describe the rate of defending and passive bystanding behavior during bullying incidents for data analysis. Chi-square test of independence was used to check the association between bystanders’ sex, their relationship with the victim and with the bully, and their reaction to the bullying incident. Thematic analysis [31] was used to analyze the qualitative data.

### Ethical considerations

Addis Ababa University School of Psychology Ethical Review Committee exempted the study from requiring ethical clearance and suggested collecting letter of permission from the school of Psychology. Accordingly, a letter of permission was collected from the School of Psychology, Addis Ababa University.

Permission letters were submitted to East Gojjam Administrative Zone Education Office. The office itself wrote a letter of permission to school directors. After receiving permission from school directors, students were also asked their willingness to participate in the study. Before data collection, informed assent and passive consent were secured from students and parents, respectively. Students were also informed that they would be free to omit any questions they did not want to answer. The participants were also informed that their identity would not be disclosed to any third party, and the information they provided would be kept confidential.

## Results

### The extent of defenders, passive bystanders, and bully supporters

Out of 511 participants who reported witnessing a single bullying incident, 55% of bystanders reported being passive bystanders, and 38% of them reported being defenders (see Table 2). The Chi-Square test revealed significant differences between the three percentages, *x*^2^ (2, N = 511) = 181.131, *p* = 0.000.

**Table 2:** Percentage of students who were involved in defending and passive bystanding behavior

Role	Frequency	Percent	*x*^2^	*p*
Passive bystander	281	55	181.131	0.000
Defender	194	38		
Bully supporter	36	7		
Total	511	100		

In the semi-structured interview, all of the participants agreed that most of the students did not want to defend the victims when witnessing school bullying. For instance, One interviewee stated, “Those who stand and watch victimization were larger than those who defend because they have the interest to see the fight and to know who wins at the end.”

### The extent of students involved in defending, passive bystanding, and bully supporting by bully-victim sex

As shown in Table 3, 39.3% of bystanders witnessed male victimizing male, 33.1% witnessed male victimizing female, 20.2% witnessed female victimizing female, and 7.4% witnessed female victimizing male.

**Table 3:** Percentage of students participating in bully supporting, defending, and passive bystanding behavior by bully-victim sex

Bully-victim sex	Supporting bully	Passive bystanding	Defending	Total
Male to male victimization	12 (6%)	114 (56.76%)	75 (37.3%)	201 (39.3%)
Female to female victimization	13 (12.6%)	57 (55.3%)	33 (32.0%)	103 (20.2%)
Male to female victimization	9 (5.3%)	88 (52.1%)	72 (42.6%)	169 (33.1%)
Female to male victimization	2 (5.3%)	22 (57.9%)	14 (36.8%)	38 (7.4%)
Total	36 (7%)	281 (55.0%)	194 (38.0%)	511 (100%)

Since the bully support role expected frequencies were less than 5 in more than 8% of the cells [32], and the purpose of the study focused on defending and passive bystanding behaviors, the bully support role was removed from further analysis (see Table 4).

**Table 4:** Chi-square analysis on bystanders’ reaction by bully-victim sex

Bully-victim sex	*N*	Choice of bystanders’ reaction		*χ*^2^	*p*
		Defending	Passive bystanding		
				1.956	0.582
Male victimizing a male	189	*75*	*114*		
Female victimizing a female	90	*33*	*57*		
Male victimizing a female	160	72	88		
Female victimizing a male	36	14	22		
Total	475	194	281		

* P* > 0.05. So, the Chi-square test shows no significant association between bully-victim sex and bystander's reaction

The Chi-Square test revealed no significant association between bully-victim sex and bystander’s reaction, χ^2^ (3, N = 475) = 1.956, *p* = 0.58, Cramer’s V = 0.06.

### The extent to which male and female bystanders defend, or remain passive upon witnessing victimization across bully-victim sex

Tables 5, 6, 7 and 8 summarizes that 67.2% of males and 32.8% females had witnessed male victimizing male, 31.2% males, and 68.8% females witnessed male victimizing femalel, 14.4% males and 85.6% females witnessed female victimizing female, and 63.9% males and 36.1% females witnessed female victimizing male.

**Table 5:** Bystander’s reaction by sex upon witnessing male victimizing male

Variables	*N*	Bystander's reaction		*χ*^2^	*p*
		Defending	Passive bystanding		
*Sex*				0.001	0.974
Male	127 (67.2%)	51 (40.2%)	76 (59.8%)		
Female	62 (32.8%)	24 (38.7%)	38 (61.3%)		
Total	189 (100%)	75 (39.68%)	114 (60.32%)

**Table 6:** Bystander’s reaction by sex upon witnessing male victimizing female

Variables	*N*	Bystander’s reaction		*x*^2^	*p*
		Defending	Passive bystanding		
*Sex*				1.881	0.170
Male	50 (31.2%)	18 (36%)	32 (64%)		
Female	110 (68.8%)	56 (49.1%)	54 (50.9%)		
Total	160 (100%)	88 (55%)	72 (45%)

**Table 7:** Percentage of students participating in defending and passive bystanding behavior upon witnessing female-on-female victimization

Variables	*N*	Bystander's reaction	
		Defending	Passive bystanding
*Sex*			
Male	13 (14.4%)	6 (46.2%)	7 (53.8%)
Female	77 (85.6%)	27 (35.1%)	50 (64.9%)
Total	90 (100%)	33 (36.67%)	57 (63.33%)

**Table 8:** Bystander’s reaction by sex upon witnessing female-on-male victimization

Variables	*N*	Bystander's reaction		χ^2^	*p*
		Defending	Passive bystanding		
*Sex*				1.057	0.304
Male	23 (63.9%)	7 (30.4%)	16 (69.6%)		
Female	13 (36.1%)	7 (53.8%)	6 (46.2%)		
Total	36 (100%)	14 (38.89%)	22 (61.11%)		

Among students who witnessed male victimizing male, 40.2% of boys and 38.7% of girls defended victims. Besides, 36% of boys and 49.1% of girls who witnessed male victimizing female helped victims in some way. Regarding students who saw female victimizing female, 46.2% of boys and 35.1% of girls defended victims. Moreover, 30.4% of boys and 53.8% of girls helped victims when witnessing female victimizing male.

The Chi-Square test revealed no significant association between bystander’s sex with victimization across bully-victim sex and bystander’s reaction. The Chi-Square test values were χ^2^ (1, N = 189) = 0.001, *p* = 0.974, *phi* = − 0.014, for students witnessing male victimizing male; *χ*^2^ (1, N = 160) = 1.881, *p* = 0.170, *phi* = − 0.122, for students witnessing male victimizing female; and χ^2^ (1, N = 36) = 1.057, *p* = 0.304, *phi* = − 0.231, for students witnessing female victimizing male.

The interview data revealed that boys and girls intervened when witnessing school bullying. For instance, Hermela noted, “When male victimizes female, mostly girls hold girls and boys hold boys.” Kidist, a ninth-grade student, also indicated, “When female victimizes female, both boys and girls may intervene.”

The qualitative data demonstrated a dissimilar intervention approach between girls and boys when witnessing male physically victimizing male. Male students, most of the time, defend directly when witnessing male physically victimizing male. On the other hand, girls can participate in defending indirectly by screaming or calling other students or reporting the case to the school authority. For instance, Hermela says, “When male physically attacks male, mostly boys and teachers directly intervene.” Debasu, an eighth-grade student said “If a girl directly intervenes when male is victimized, rumors will spread which show the girl has love affair with the victim.”

### The extent of students’ participation in defending and passive bystanding behavior by relationship with the victim or bully

As indicated in Tables 9 and 10, bystanders were asked to report their relationship with victims and bullies. Among those who reported their relationship with victims and bullies, 3.6% and 3.8% reported to be relatives, 26.7% and 11.6% close friends, 24.6% and 24.2% classmates, 24.6% and 26.3% knew the victim/bully, but have no close relationships, and 20.4% and 34.1% did not know the victim and bully, respectively. Among those who reported their relationship with the victim, 52.9% of relatives, 60.6% of close friends, and 47.8% of classmates defended the victim. Similarly, among those who reported their relationship with the bully, 61.1% of relatives, 49.1% of close friends, and 47% of classmates defended the victim.

**Table 9:** Bystander’s reaction by relationship with the victim

Relationship with the victim	*N*	Bystander's reaction		*x*^2^	*p*
		Defending	Passive bystanding		
				32.79	0.000
Relative	17 (3.6%)	9 (52.9%)	8 (47.1%)		
Close friend	127 (26.7%)	77 (60.6%)	50 (39.4%)		
Classmate	117 (24.6)	44 (47.8%)	73 (69.2%)		
Person that I knew	117 (24.6%)	36 (30.8%)	81 (69.2%)		
Person that I didn’t know	97 (20.4%)	28 (28.9%)	69 (71.1%)		
Total	475 (100%)	194 (40.8%)	281 (59.2%)

**Table 10:** Chi-square analysis in bystander’s reaction by relationship with the bully

Relationship with the victim	*N*	Bystander's reaction		*χ*^2^	*p*
		Defending	Passive bystanding		
				9.847	0.043
Relative	18 (3.8%)	11 (61.1%)	7 (38.9%)		
Close friend	55 (11.6%)	27 (49.1%)	28 (50.9%)		
Classmate	115 (24.2%)	54 (47.0%)	61 (53.0%)		
Person that I knew	125 (26.3%)	43 (34.4%)	82 (65.6%)		
Person that I didn’t know	162 (34.1%)	59 (36.4%)	103 (63.6%)		
Total	475 (100%)	194 (40.8%)	281 (59.2%)		

The Chi-Square test revealed that there is a significant association between the relationship with the victim and bystander’s reaction, χ^2^ (4, 475) = 32.79, *p* < 0.001, phi = − 0.263; and between relationship with the bully and bystander’s reaction, *χ*^2^ (4, N = 475) = 9.847, *p* = 0.043, *phi* = − 0.114.

The qualitative data through interview indicated that bystanders’ close relationship with the victim or/and bully as key determinant of defending upon witnessing school bullying. For instance, Debasu said “I have entered (involved in defending) because both the perpetrators and the victims were my friends.” A grade eight student named Binyam stated, “Students who are relative or close friends…to the victim/bully would not have any role other than separating the bully and the victim.” Hermela also noted that relatives, friends, and teachers are defenders during victimization.

On the other hand, not being a friend of the bully or the victim was reported as a possible reason for bystanders’ passive bystanding. For instance, Hermela mentioned “bystanders’ not being the friend of the bully or the victim as one reason for bystanders to surround and watch bullying events. Had the bystanders been friends of the victim/bully, they would have intervened or they would have called a teacher.”

## Discussion

### The extent to which students defend or passively watch during bullying incidents

The findings of this study revealed that a larger proportion of students remained passive upon witnessing school bullying. Fifty five percent of bystanders were involved in passive bystanding behavior, and 38% of them involved in defending behavior.

The interview data also supported the findings of the quantitative data. All participants of the interview reported that many bystanders most often stood by passively, and only some of them defended the victim. Many participants concisely stated that when students in school witness bullying incidents, most of them often stand and observe while a small number of others decide to defend.

These findings are consistent with prior studies [14, 17]. For instance, a study conducted on college students who recalled bullying events occurring in junior high school and high school students with the same method reported that 59% of bystanders chose to remain passive upon witnessing bullying situations, and 31% of them were involved in defending on behalf of the victims [17]. Similar findings were also reported in an observational study conducted in two Toronto school children in Canada [14]. Even the percentages are very close to the ones this study found.

There are various explanations attributed to the surpassing of passive bystanders to defenders in East Gojjam Administrative Zone. One reason for passivity of bystanders during bullying incidents may involve the gradual decline of helping relationships due to urbanization. In the past, people do not often standby and watch when one individual victimized another. Findings in Yetmen, East Gojjam, revealed that when conflicts arise within and between households, they were usually resolved by neighbors. If neighbors cannot solve the problem, relatives of the two parties consider the problem and try to address it. If this level of conflict resolution fails, the elder of the community get involved [28]. So, helping each other during an emergency was the norm. Due to urbanization, the norms of helping relationships are changing somehow in the current East Gojjam. Another possible explanation for more passive bystanders to defenders involves fear of revenge. If the perpetrator is older and/or physically stronger than the bystander, the bystanders are more likely to remain passive. Student bystanders may believe that defending on behalf of the victim could lead the older/or stronger bully to attack the defender later. Many other personal and situational factors (e.g., lower level of bystander’s self-efficacy, empathy, lower number of close friends, bullying experiences, high moral disengagement) may also be used to explain greater proportions of passive bystanders to defenders in bullying situations [17, 20, 22, 26, 33].

### The extent to which male and female bystanders defend, or remain passive upon witnessing victimization across bully-victim sex

The quantitative findings demonstrated that there were no significant difference between boys and girls in defending and passive bystanding behaviors upon witnessing a boy victimizing a boy, a boy victimizing a girl, and a girl victimizing a boy.

According to the interview data, both boys and girls can intervene when a boy victimizes a boy. But, their style of intervention may differ. Boys may intervene directly when witnessing physical bullying, whereas girls may intervene indirectly. Many participants said that boys, teachers, and adults directly intervene when a boy physically victimizes a boy. One possible reason for the direct intervention of more boys than girls was that if a girl intervenes directly when a boy victimizes a boy, rumors of love between the girl and the victim will spread. In the culture of the study area, having a boyfriend for a girl and a girlfriend for a boy is not a commonly accepted norm at that age level. If they establish such kinds of friendship, they do not disclose it to others. If other students know the relationship, they become the target of the rumor. So as to avoid being the target of the rumor, the girl will decide to use indirect strategies to help the victim.

Another possible explanation for more direct defending of boys than girls in physical bullying was that boys were more often socialized and culturally expected to defend directly than girls. Let alone defending on behalf of the victim, boys are expected to be a winner in any fight by their families and are not accepted by families if beaten up by anyone. If they fail to win the fight, their parents could further beat them. Though girls’ involvement in separating the bully and the victim is less direct, they frequently call defenders by screaming.

The finding also indicated that when a boy victimizes a girl, a girl victimizes a girl, and a girl victimizes a boy, most of the interview participants reported that both boys and girls are engaged in defending. This finding partly contradicts some other findings [24, 25]. To explain these findings further, future researches are needed.

#### Does the relationship between the bystander and the victim or the bystander and the bully make a difference in the bystander’s reaction?

The current study revealed that students who were reported to be close friends, classmates, and relatives of the victims appear to defend the victim more than persons who either knew the victim or did not know them. Consistent with the current study, five studies included in one systematic review have examined the association between friendship with students involved in bullying situations and defending [33]. The studies revealed that youth were more likely to defend when the victimized youth was their friend, relative to a neutral peer. Similarly, some studies [26, 27] revealed the association between bystanders’ close relationship with the victim and helping. For example, suppose a bystander is watching one’s own friend being bullied. In that case, the situation evokes more distressing emotions of empathy, sympathy, guilt, or anger and a stronger moral obligation and responsibility to intervene to help one’s friend [27].

The findings from the interview data also corroborated the quantitative results. The study showed that after bystanders witnessed bullying incidents, they evaluate their relationships (friendship, kinship, and disliking) with the bully, victim, or both before deciding to defend or passively watch the bullying incident. If bystanders witness victims with intimate relationships (friendship and blood relationship), they are more likely to defend the victim. Participants mentioned being close friends, relatives, and teachers with the victim as contributing factors to defending.

The finding that students who were reported to be relatives, close friends, and classmates of the bully appear to defend the victim more than persons who know and those who did not know the bully was unexpected. The qualitative interview also supported this finding. Some interview participants disclosed that having a close relationship with the bully would motivate the bystander to assist the victim. If bystanders are close friends or relatives of the bully, they can enter with confidence to protect the victim believing that the bully will not attack them later. Another possible reason for bystanders who have close relationships with the bully to stop the bully could be the belief that the problem will worsen and affect the whole family and its relatives. However, one participant reported that if bystanders have a close relationship with the bully, they might assist the bully to harm the victim further. Thus, further studies are needed.

### Limitations of the study

The current study has some limitations. First, the study participants were limited to young and middle adolescents in East Gojjam Administrative Zone. This could reduce the diversity of the sample and the generalizability of the findings. Had I included adults as well, the findings could have been more generalizable. Second, the quantitative and qualitative findings on defending and passive bystanding behaviors were based on self-report measures. In self-reporting data, study participants may not always provide honest evidences. Third, the current research was cross-sectional, where cause and effect relationships could not be inferred.

Fourth, it is expected that if the perpetrator is older and/or physically stronger than the bystander, the bystander is more likely to remain passive during the incident of bullying. However, the current study did not collect information on age and/or physical differences between bully and bystander. If future studies include age and physical differences between the bystander and the bully, it would have more insights into school bullying literature.

## Conclusion

Practice of defending among students attending their education in governmental primary and secondary schools in East Gojjam Administrative Zone was low. Close social relationships (being close friends, relatives, and classmates) with the victim and bully were significantly associated with the practice of defending. The findings of our qualitative study also showed that the number of passive bystanders was larger than defenders during witnessing school bullying; and bystanders’ close relationship with the victim, or/and bully as key determinants of defending.

High prevalence of passive bystanding behavior demand prevention programs that can discourage bullying in schools among bystanders in bullying situations through encouraging defending behavior irrespective of bully-victim sex, and helping bystanders establish close social relationships with the victim or/and bully.

## Data Availability

The datasets that support the findings of this study are not publically available at present. The authors need to use the data for further works before data could be made available. Besides, we have not received consent from participants to share the data on the web but, will be available from the corresponding author on reasonable request.
